# Features of Child Food Insecurity after the 2010 Haiti Earthquake: Results from Longitudinal Random Survey of Households

**DOI:** 10.1371/journal.pone.0104497

**Published:** 2014-09-10

**Authors:** Royce A. Hutson, Eileen Trzcinski, Athena R. Kolbe

**Affiliations:** 1 School of Social Work, Wayne State University, Detroit, Michigan, United States of America; 2 School of Social Work and Department of Political Science, University of Michigan, Ann Arbor, Michigan, United States of America; Vanderbilt University, United States of America

## Abstract

**Background:**

Recent commentary on the health consequences of natural disasters has suggested a dearth of research on understanding the antecedents prior to the disaster that are associated with health consequences after the disaster. Utilizing data from a two-wave panel survey of Port-au-Prince, Haiti, conducted just prior to and six weeks after the January 2010 earthquake, we test factors prior to the quake hypothesized to be associated with food insecurity after the quake.

**Methods:**

Using random Global Positioning System (GPS) sampling, we re-interviewed 93.1% (N = 1732) of the original 1,800 households interviewed in 2009. Respondents were queried with regard to mortalities, injuries, food security, housing, and other factors after the quake.

**Findings:**

Child food insecurity was found to be common on all three indices of food security (17.2%–22.6%). Additionally, only 36.5% of school-aged children were attending school prior to the quake. Findings suggest that prior schooling was associated with a substantial reduction on food insecurity indices (OR 0.62–0.75). Findings further suggest that several household characteristics were associated with food insecurity for children. Prior chronic/acute illnesses, poor living conditions, remittances from abroad, primary respondent mental health, and histories of criminal and other human rights violations committed against family members prior to the quake were associated with food insecurity after the earthquake. Earned household income after the quake was only associated with one of the measures of food insecurity.

**Interpretation:**

Food insecurity for children was common after the quake. Those households vulnerable on multiple dimensions prior to the quake were also vulnerable to food insecurity after the quake. Remittances from abroad were leading protective factors for food security. Because Haiti is well known for the potentiality of both hurricanes and earthquakes, reconstruction and redevelopment should focus on ameliorating potential vulnerabilities to poor outcomes in these natural disasters.

## Introduction

In January 12, 2010, an earthquake registering 7.0 on the Richter scale ravaged the island nation of Haiti. The official government estimates of mortality after the quake ranged from 220,000–250,000. Research conducted by Kolbe, et al. [1] found that approximately 160,000 died in Port-au-Prince alone either during or in the six weeks after the quake. Almost 11,000 women and girls were sexually assaulted in the quake's aftermath.

Although the destruction and human toll that this earthquake created has been well reported, what has not been given attention are the risk factors that were associated with victimization in this quake. Little is known about what socio-demographic characteristics are associated with health consequences as there is scant literature on the subject. Noted publications such as the Lancet and Nonprofit Quarterly [2] [3] have recently focused attention on this lack of knowledge, suggesting that little rigorous research has been conducted regarding antecedents to poor outcomes. These publications have suggested that rigorous research could lead to proper planning and development, which could then ameliorate the mortality and morbidity that occurs as a result of future natural disasters. This is of particular importance as Ambraseys and Bilham, [4] suggest that governmental corruption that resulted in poor structural quality of buildings may to be blame for the significant death toll. Indeed, they note that an earthquake of similar magnitude, which occurred in New Zealand, resulted in no fatalities. To make matters even more urgent, there is considerable likelihood that another earthquake is possible in the near future in Haiti. Recent research suggests that the tectonic strain at the fault line responsible for the quake remains strained [5].

There has been some work on this disaster. Kolbe et al. [1] found that children were significantly more likely to have been killed during the quake or in the six weeks after the quake. Compared with adults, children under the age of 18 were six times more likely to have died during the quake, 11 times more likely to have died of injuries immediately following the quake, and twice as likely to have died as a result of illness. The authors also found that those who received any remittances from abroad were 35 times more likely to be food secure, as rated by the USDA, relative to those who received no remittances.

In this paper, we examine the association between individual- and household-level characteristics as it relates to two important child outcomes, schooling and food security. To note the relative vulnerability of children before the quake, we examine schooling prior to its occurrence and then examine how schooling, living conditions, abuse history, and socio-demographic factors relate to food security after the earthquake. We test the hypothesis that pre-quake enrollment in schooling is associated with material hardship, living conditions, abuse history, and socio-demographic characteristics. Secondly, we test the hypothesis that household damage after the quake was associated with the aforementioned factors. Lastly, we test the hypothesis that school enrollment along with housing damage from the quake were associated with food security after the quake.

### Conceptual Framework: Sen's Theory of Entitlement Exchange, Capabilities, and Functionings

The conceptual framework used in this article draws primarily from the theories of Amartya Sen. The framework of Sen, [6] has been drawn upon extensively to reframe how the sources of food insecurity are identified and how short and long term policy responses should be formulated to alleviate food insecurity. As noted by Webb et al. [7], Sen's principal thesis centered on the fact, “that people often fall prey to food deprivation no so much because food is unavailable but rather because their access to such food is constrained (p. 1404S)”. In Sen's framework, lack of access to food stems not only from poverty, but also from a lack of entitlements that provides households with adequate access to food. For children, the set of entitlements that they currently experience will largely be determined by the capabilities achieved by adults within their household as children themselves have limited resources to achieve food security [8]. Natural disasters can clearly alter the household's ability to achieve food security. However, the extent to which natural disasters affect this ability will likely depend, at least in part, on pre-disaster entitlements and capabilities. Prior research following Hurricane Katrina in the US showed disparate negative impacts by race and class [10], [11].

In our own conceptual model, we examine the extent to which a range of characteristics of the children, the adult respondent, and the household function as predictors of whether children attended school prior to the earthquake and whether they experienced food insufficiency following the earthquake. Our purpose was to test the extent to which vulnerabilities experienced by children before and after natural disasters are associated with particular sets of predictors. Our research can thus contribute to the body of research which indicates that negative and sometimes horrific consequences of natural disasters are neither random nor completely unpreventable [9], [12]; A specific contribution of our work is its focus on the multiple dimensions of vulnerabilities of children, including different measures associated with poverty and with violence. For this reason, we also use adult and household predictors for both outcomes that pertain to pre-earthquake adult and household circumstances. If pre-earthquake variables predict negative outcomes post-disaster, this evidence would provide additional support for the need to address economic and social inequities and broad-scale issues of development before disasters. Within our model, we predict that the following variables will be associated with the probability that a child attend school and that a child experiences different degrees of food insufficiency.

#### The Experience of Violence

We hypothesize that violence and the direct experience of physical or sexual assault will diminish the capabilities of both children and adults in the household, subsequently the likelihood of food insecurity will increase. Another predictor that we model is the perceptions of crime and insecurity within the community which we hypothesize will result in less food security.

#### Household Resources and Quality of Housing

Household income is hypothesized to be positively associated with whether the child attends school and negatively associated with whether the child experiences food insufficiency. Contributions of the child to household income via child labor, however, are expected to reduce the likelihood that the child attends school.

Remittances from friends and relatives are expected to play a larger positive role than other sources of income in increasing the probability that a child attends school and in reducing the probability of food insufficiency. There are two rationales for this hypothesis. First, household income that is attained through the labor market demands a commitment of time and energy that will reduce the time available for supervision required by children who attend school. Additionally, monies and aid received from governmental agencies and NGOs can require large time commitments on the part of the recipients and the imposition of restrictions on how the aid can be used. Secondly, remittances from abroad require little time or energy commitments on the part of the recipients; nor are remittances typically restricted as to how the monies can be used.

#### Health

We hypothesize that children who experience acute or chronic illness will be less likely to attend school, but also less likely to experience food insufficiency than children without chronic or acute illnesses. In households in which children are already suffering from acute or chronic illness, we hypothesize the adults members of the household will attempt to make additional sacrifices to ensure that the health of the children does not continue to deteriorate. We also hypothesize that adult members of the household who are also suffering from acute or chronic illness will have fewer resources available to ensure that the child attends school and that the child has adequate access to food.

## Methods

A series of two cohort surveys were conducted in the greater Port-au-Prince area just prior to and six weeks after the earthquake. This initial 2009 baseline survey was administered in the Port-au-Prince metropolitan area as part of a wider University of Michigan study on health and human rights violation experiences in Haiti. In 2005, Kolbe & Hutson [13] conducted a survey on human rights and other criminal violations in Port-au-Prince in the almost two-year period following the ouster of Jean Bertrand Aristide from the presidency. The 2009 survey was conducted in order to establish whether living conditions and security had improved under the Preval presidency.

To obtain a proper sampling frame for this survey, research team members mapped the geographic boundaries of the area, using GPS locators. The team established and mapped the borders of the metropolitan area; then determined the borders of specific neighborhoods. A list of randomized GPS coordinates within the metropolitan area was then created. Two- or three-person interviewing teams then visited each location, identified all households within 20 m of the GPS coordinate, and randomly selected one, using a random number table. If there were no households within 20 m, a replacement household was identified, using the same procedure. The team visited each household up to four times in order to contact an adult household member. In 110 cases an adult household member was not located in any of the four visits. To randomize the adult respondent, the adult with the most recent birthday was selected. A description of the study was read to each respondent and a handout describing the study was also provided. This sampling method has been used in several locations by the authors in other disaster and conflict zones [1], [6], [14].

A total of 1,500 households were randomly selected and interviewed from the Port-au-Prince area during the 2009 fielding. In order to get proper representation in the survey from the more densely populated “popular zones,” an additional 300 households were over-sampled (100 per neighborhood) in the three urban zones of Martissant/Gran Ravine, Cite Soley, and Bel Air. This resulted in a total sample of 1,800 households. The zones selected for over-sampling all featured proportionately high rates of crime and human rights violations in a previous study [13]. The sampling procedures that were utilized in the overall sample were also employed for the over-sampled zones. Of all households, 82 refused to participate in the study. Taking into account refusing households and those in which no adult was found at home after four visits, the response rate for Port-au-Prince households in the baseline survey was 90.3%.

### Post-Earthquake Survey

Between 18 February and 4 March 2010, the research team attempted to contact the 1,800 Port-au-Prince households that had been surveyed in late 2009. Most households were located in or near their previous location. In cases in which they were not located nearby, survey team members interviewed remaining residents about the location of the missing household. When this did not lead to the location of a household, we discussed with local community leaders the likely location of the household. Survey teams then went to ask nearby residents in the likely location if the displaced household had located in their community. Often times, this was in an IDP camp. Many IDP camps maintained lists of residents within the camps. As most households that were not located in their home resided in either formal or informal IDP camps, these lists proved invaluable in locating households. Research teams went throughout the country as well as into the Dominican Republic to locate and interview respondents. Extraordinary efforts were made to locate each household, with interviewers traveling by bus, taxi, on foot and by donkey to remote villages and large cities. In-person interviews of displaced respondents were also conducted in South Florida, New York, Boston, and Montréal. Not all respondents could be physically reached and 52 respondents were interviewed by telephone. No incentives were provided to the respondents.

A household member (in most cases, the original respondent) was located in 1,732 (93.1%) cases. However, in some households, the original respondent had relocated to a different area, while other household members remained in the same neighborhood. In these cases an adult remaining in the neighborhood was randomly selected for the follow-up interview. In 91 (5.1%) cases the original respondent was unavailable for other reasons (death, illness, or other personal reasons). In these latter cases, another adult household member was randomly selected. A full description of the sampling methodology used and of efforts to locate households is outlined in Kolbe et al. [1].

#### Survey Design and Variable Definitions

Both surveys were designed to collect individualized information on the respondents and other household members. In addition, aggregate household information was also collected.

#### Child Labor and Education Variables

Demographic information was collected using sections from the International Labor Organization's (ILO) National Child Labor Study [15].

#### Food Insufficiency

The United States Department of Agriculture's (USDA) Household Standard Food-Security/Hunger Survey [9] was used to measure levels of hunger and need. This instrument has been widely used to assess hunger and food security in the United States and elsewhere. The original intention of the instrument was to assess the food security of a survey population in the prior year. Following guidelines from the USDA, the wording was changed to capture experiences “since the earthquake” rather than “in the last 12 months.” The USDA Household Survey has been used extensively not only within the United States, but also within many developing countries, including Haiti. [7]; [16], [17] [18]. In this study, we limited the analysis to three items from the scale that specifically pertained to food insufficiency for children in the household.

#### Assaults

Crime victimization history for sexual and physical assaults was obtained using the Human Rights History, a structured interview previously applied in Haiti and Lebanon [14], [13]. If there was a death in the household, information on the date of death and the perceived cause of death were queried. In the case of possible homicide, the perceived perpetrator, method of killing, and location of the murder was recorded. Information on victimization was collected for every member of the household. Specifically, each respondent was asked to answer the following questions for him/herself and for every member of the household, including both adults and children:

In the past year, has anyone physically attacked you (hurt your physical body)?

What was the date of the last incident?

How were you injured? (if multiple injuries, list most severe).

Each respondent was also asked whether he/she had been sexually assaulted in the past year and whether each individual member of the household, including children, had experienced a sexual assault.

#### Household Resources and Quality of Housing

The survey obtained information on household income, remittances received, and replies to a number of specific questions about the dwelling in which the household lived. For the 2010 survey, questions were added to address access to basic needs, including security, food, water, health services and housing. In the analysis below, we included controls for household income, the amount of remittances received, and a set of dummy variables indicating whether the dwelling had electricity, flush toilets, or running water. We also controlled for the respondent's assessment of the level of insecurity and crime experienced by the household prior to the earthquake.

#### Post-Traumatic Stress

Post-traumatic Stress Disorder (PTSD) symptomology in 2009 was assessed using the PTSD section of the Harvard Trauma Questionnaire (HTQ). The instrument has not been formally tested in Haiti, As a result, we did not use this instrument as a diagnostic tool but as a continuous measure of symptomology to access the relative psychological health between respondents. With that, the instrument has been utilized in multiple contexts and has demonstrated strong reliability and validity [19].

#### Other Demographic Information

The survey collected information on age and number of all household members, on the health of each household member, and on the working status and educational level. In the analysis below, the educational level of the respondent is used as a control variable. We also constructed a variable that indicated the percentage of household members who were ill or chronically ill prior to the earthquake.

The final survey was translated into Creole and pilot-tested with 112 Haitian citizens prior to fielding. For the sake of analysis in this study, we focused on the three food security queries that related directly to child food insecurity (See Appendix I in File S1 for post-quake survey).

#### Ethics Review and Conflict of Interest

The survey was approved by the University of Michigan Institutional Review Board. Participants gave verbal informed consent. Given safety concerns regarding identifying information on the survey forms, written consent was not obtained. The University of Michigan IRB approved this consent procedure. Interviewers documented verbal consent as refusals were documented as such on the survey form.

#### Data Analysis

The sample was weighted according to Haitian census data collected in 2003 and extrapolated by the statistics office in Haiti to 2009 [20]. Each household was assigned to an administrative unit (commune), according to the location of the household before the earthquake. Taking the estimated population size of each commune for 2009, each individual respondent was weighted up to represent a proportion of the population size for that commune. The population estimate for the greater Port-au-Prince metropolitan area for this sample was 2,713,599. Data were analyzed using Stata Version 11. All analyses were weighted and estimated with robust standard errors, in which the household was the cluster variable.

## Results

Table 1 provides descriptive statistics for the sampled households. As we believed that earthquake damage to the home would be a substantial predictor of food security we examined the likelihood of several socio-demographic characteristics on whether a residence had visible damage as a result of the earthquake. Table 2 presents the logistic regression results of household-level predictors of whether the household demonstrated no visible damage following the earthquake. Because logistic regressions estimate odds ratios that do not provide direct information on the underlying probabilities, we provide predicted probabilities in Appendix II in File S1 for visible damage. These predicted probabilities are the marginal predicted probabilities for each category, based on the estimated coefficients provided in Table 2.

**Table 1:** Descriptive Statistics, Children Aged 6 to 17, Weighted Percents and Means.

Variable		Estimate	95% Confidence Interval	
			Lower	Upper
Child was attending school prior to the earthquake	No	63.5%	61.1%	65.9%
	Yes	36.5%	34.1%	38.9%
In the last month, child was hungry	No	82.8%	80.0%	85.3%
	Yes	17.2%	14.7%	20.0%
In the last month, the child skipped meals	No	77.5%	74.4%	80.3%
	Yes	22.5%	19.7%	25.6%
In the last month, did you cut the size of children's meals	No	77.4%	74.3%	80.2%
	Yes	22.6%	19.8%	25.7%
Child was victimized prior to the earthquake	No	95.9%	95.2%	96.4%
	Yes	4.1%	3.6%	4.8%
Child was chronically or acutely ill before earthquake	No	79.6%	78.0%	81.2%
	Yes	20.4%	18.8%	22.0%
Gender	Boy	47.3%	45.4%	49.2%
	Girl	52.7%	50.8%	54.6%
Child had earned income	No	79.6%	78.7%	80.4%
	Yes	20.4%	19.6%	21.3%
Age of child before earthquake		11.37	11.23	11.50
Electricity, toilets, running water	None	17.9%	15.3%	20.8%
	One	59.8%	56.3%	63.2%
Respondent had less than a high school education	No Yes	27.8% 72.2%	24.8% 69.1%	30.9% 75.2%
Household was victimized at least once before earthquake	No	81.3%	78.2%	84.0%
	Yes	18.7%	16.0%	21.8%
Log of household income		2.9142	2.8643	2.9641
Log of monetary gifts received by household		.9663	.8590	1.0735
Percentage of household members who were chronically or acutely ill prior to earthquake		.208	.195	.221
PTSD Mean Score		1.79	1.73	1.84
Respondent rated level of insecurity or crime before earthquake as very serious/serious	No	16.3%	13.9%	19.1%
	Yes	83.7%	80.9%	86.1%
Number of children in household		5.19	5.03	5.35
Number of adults in household		2.75	2.65	2.85
House had no visible damage following earthquake	No	63.4%	60.0%	66.6%
	Yes	36.6%	33.4%	40.0%
Unweighted Number of Cases	3,596			

**Table 2:** Predictors of Whether the House had No Visible Damage following the Earthquake.

VARIABLES	Odds Ratio	Standard Error	95% Confidence Interval
Ln Gifts	1.15**	(0.05)	1.06–1.26
Household had no or one only of electricity, flush toilet, or running water	0.70*	(0.10)	0.52–0.93
Education of Respondent: More than High School	1.66+	(0.46)	0.96–2.85
Top income quartile	1.44*	(0.22)	1.06–1.95
Constant	0.87	(0.13)	0.65–1.17
Observations	3,676		
Pseudo R	0.02		
p	0.001***		
chi2	22.41		
d.f.	4		
Number of clusters	1342		

***p<0.01,

**p<0.05,

*p<0.1, +p<.10.

Table 3 presents logistic regression results that predict whether the child or adolescent was attending school prior to the earthquake. As with the logistic regression analysis with home damage, because logistic regressions estimate odds ratios that do not provide direct information on the underlying probabilities, we provide predicted probabilities in Appendix III in File S1 for children with different characteristics. Table 4 shows logistic regression estimates for whether the child went hungry after the earthquake estimated with and without the household damage variable.

**Table 3:** Logistic Regression of the Probability that Child or Adolescent Aged 6 to 17 Was Attending School Prior to the Earthquake.

	Odds Ratio (Robust std. error)		95% Confidence Interval
Child was victimized	0.55	**	(0.324–0.945)
	(0.151)		
Child had a chronic or acute illness	0.73	**	(0.569–0.945)
	(0.095)		
Female	0.47	***	(0.393–0.556)
	(0.042)		
Child had earned income	0.55	***	(0.398–0.760)
	(0.091)		
Age of Child	1.02		(0.995–1.048)
	(0.014)		
Log household income	1.73	***	(1.165–2.575)
	(0.350)		
Log monetary gifts received by household	1.55	***	(1.437–1.671)
	(0.060)		
Household had no electricity, flush toilet, or running water	0.80		(0.594–1.069)
	(0.119)		
Percentage of household members who were chronically or acutely ill	0.25 (0.101)	***	(0.114–0.553)
Household had no electricity, flush toilet, or running water	0.80 (0.119)		(0.594–1.069)
			
Household respondent has less than a high school education	0.78 (0.093)	**	(0.619–0.985)
Household respondent rated level of insecurity as serious	0.71	***	(0.547–0.913)
	(0.092)		
Number of children in household	1.02		(0.974–1.060)
	(0.022)		
Number of adults in household	0.95		(0.882–1.015)
	(0.034)		
Constant	0.21	**	(0.050–0.863)
	(0.151)		
Wald chi2(13) = 487.15***			
Pseudo R2 = 0.19			
Observations = 3,596			

***p<0.01,

**p<0.05,

*p<0.1.

**Table 4:** Logistic regression of the probability a child was hungry with and without household damage.

	Column 1	Child was hungry	Column 3	Child was hungry	Chow Test of Equality of Coefficients
	Child was hungry, without variable of household damage	95% CI	Child was hungry, with variable of household damage	CI	Column 1–Column 3
VARIABLES	Odds Ratios	Odds Ratio	Odds Ratio	Odds Ratio	p level
Age of Child	0.97	0.94–1.01	0.98	0.94–1.01	chi2(1) = 0.40
	(0.02)		(0.02)		Prob>chi2 = 0.5276
Female	0.95	0.74–1.21	0.95	0.74–1.21	chi2(1) = 0.01
	(0.12)		(0.12)		Prob>chi2 = 0.9392
Child Was in School	0.63**	0.47–0.84	0.64**	0.48–0.85	chi2(1) = 0.02
	(0.09)		(0.09)		Prob>chi2 = .8867
Child suffered from chronic/acute illness pre earthquake	0.85	0.68–1.07	0.85	0.68–1.07	chi2(1) = 0.02
	(0.10)		(0.10)		Prob > chi2 = 0.8922
Log of Household Income	0.95	0.78–1.17	0.96	0.77–1.18	chi2(1) = 0.00
	(0.10)		(0.10)		Prob>chi2 = 0.9838
Household had electricity, flush toilets, running water					
No	8.20***	3.54–18.96	8.19***	3.56–18.82	chi2(1) = 0.35
	(3.51)		(3.48)		Prob>chi2 = 0.5553
One only	2.80**	1.30–6.00	2.75**	1.28–5.91	chi2(1) = 0.42
	(1.09)		(1.07)		Prob>chi2 = 0.5158
Log of Monetary Remittances Received by Household	0.80*	0.67–0.95	0.79*	0.66–0.95	chi2(1) = 0.03
	(0.07)		(0.07)		Prob>chi2 = 0.8528
Percentage of household members who were chronically/acutely ill	4.91**	1.78–13.56	4.97**	1.76–14.05	chi2(1) = 0.37
	(2.54)		(2.63)		Prob>chi2 = 0.5450
Number of children in household	0.85***	0.77–0.92	0.85***	0.77–0.93	chi2(1) = 0.01
	(0.04)		(0.04)		Prob>chi2 = 0.9326
Number of adults in household	0.80**	0.70–0.92	0.80**	0.70–0.92	chi2(1) = 0.28
	(0.06)		(0.06)		Prob>chi2 = 0.5953
Household respondent level of PTSD symptoms	1.53*	1.10–2.11	1.51*	1.09–2.09	chi2(1) = 0.33
	(0.25)		(0.25)		Prob>chi2 = 0.5649
Household member was victimized pre earthquake	2.07*	1.14–3.77	2.11*	1.16–3.85	chi2(1) = 1.69
	(0.63)		(0.65)		Prob>chi2 = 0.1937
Household respondent viewed level of insecurity as serious pre earthquake	2.11*	1.08–4.12	2.20*	1.12–4.34	chi2(1) = 0.35
	(0.72)		(0.76)		Prob>chi2 = 0.5553
House had no visible damage following earthquake			0.54**	0.35–0.84	
			(0.12)		
Constant	0.06***	0.01–0.26	0.07***	0.02–0.33	
	(0.04)		(0.06)		
					
Observations	3,596	3,596	3,596	3,596	
Pseudo R^2^	0.233		0.242		
N_clust	1300		1300		

***p<0.01,

**p<0.05,

*p<0.1.

Table 5 provides the logistic regression findings of having to cut the size of children's meals and having to skip meals after the quake. In Appendix IV in File S1, the underlying predicted probabilities of either being hunger, skipping meals and cutting the size of meals by; 1) whether the child was in school prior to the quake, 2) whether there was visible damage after the quake, 3) whether the household prior to the quake had electricity, a flush toilet or running water, 4) whether the household was in the lowest income quartile, 5) whether the household had no remittances or were in the top quartile of remittances received after the quake, 6) whether someone in the household had been victimized prior to the quake, 7) whether the perceived security situation was serious or not, 8) whether no one in the household was acutely ill or over 50% were prior to the quake is presented. Lastly, Appendix V in File S1 shows a test of the equality of coefficients for the three food security variables stratified by the child in school prior to the quake.

**Table 5:** Logistic Regression of Cutting the Size of Children's Meals.

	Cutting the Size		Skipped Meals	
VARIABLES	Odds Ratio	95% Confidence Interval	Odds Ratio	95% Confidence Interval
	(Std. Error)	Odds Ratio	(Std. Error)	Odds Ratio
Age of Child	1.00	0.97–1.03	0.98	0.95–1.01
	(0.02)		(0.02)	
Female	0.89	0.72–1.10	0.83+	0.66–1.03
	(0.10)		(0.09)	
Child Was in School	0.75*	0.57–0.98	0.62***	0.48–0.80
	(0.10)		(0.08)	
Child suffered from chronic/acute illness pre earthquake	0.92	0.75–1.14	0.89	0.72–1.10
	(0.10)		(0.10)	
Log of Household Income	0.92	0.77–1.10	0.97	0.80–1.18
	(0.08)		(0.10)	
Electricity, Flush Toilet, Running Water				
No	7.45***	3.72–14.91	10.25***	4.90–21.41
	(2.64)		(3.85)	
One only	2.23*	1.18–4.22	3.16***	1.63–6.11
	(0.72)		(1.06)	
Log of Monetary Remittances Received by Household	0.71***	0.58–0.87	0.81**	0.69–0.95
	(0.07)		(0.07)	
Percentage of household members who were chronically/acutely ill	9.92***	3.80–25.86	8.91***	3.16–25.11
	(4.85)		(4.71)	
Number of children in household	0.87**	0.80–0.95	0.88**	0.81–0.95
	(0.04)		(0.04)	
Number of adults in household	0.79***	0.69–0.90	0.82**	0.72–0.94
	(0.05)		(0.06)	
				
Household respondent level of PTSD symptoms	1.65***	1.24–2.20	1.76***	1.31–2.35
	(0.24)		(0.26)	
Household member was victimized pre earthquake	2.23**	1.32–3.78	1.63+	0.94–2.85
	(0.60)		(0.46)	
Household respondent viewed level of insecurity as serious pre earthquake	2.10*	1.06–4.18	2.67**	1.44–4.95
	(0.74)		(0.84)	
House had no visible damage following earthquake	0.68+	0.46–1.00	0.58**	0.39–0.85
	(0.14)		(0.11)	
Constant	0.07***	0.02–0.27	0.04***	0.01–0.16
	(0.05)		(0.03)	
				
Observations	3,596	3,596	3,596	3,596
Pseudo R^2^	0.271		0.264	
Number of clusters	1300		1300	

***p<0.01,

**p<0.05,

*p<0.1.

### Descriptive Statistics for Children Aged 6–17

Reviewing Table 1, of children, 52.7% (CI 50.8–54.6) were female. The average age of children was 11.37 (CI = 11.23–11.50). Of children, 20.4% (CI 19.6–21.3) were reported to having earned income prior to the quake. We find that 36.5% (95% CI 34.1–38.9) of the children were attending school prior to the quake. With regards to physical and sexual assault, 4.1% (CI = 3.6–4.8) of children were reported to have been assaulted prior to the quake.

Of households, 36.6% (95% CI 33.4–40.0) reported that they had visible damage to their homes. With regards to food security, 17.2% (95% CI 14.7–20.0) reported that their child had went hungry in the previous month; 22.5% (95% CI 19.7–25.6) stated that their child had skipped a meal in the past month; and 22.6% (95% CI 19.8–25.7%) reported that they had cut the size of their children's meals in the previous month.

### Predictors of Whether the House Had No Visible Damage following the Earthquake

From Table 2, the amount of remittances (OR = 1.15, CI = 1.06–1.26), whether the main respondent had more than a high school diploma (OR = 1.66, CI = 0.96–2.85), and being in the top income quartile (OR = 1.44, CI = 1.06–1.95) were significantly associated with a greater likelihood of not having experienced earthquake damage to the home. Having only one or no amenities (electricity, flush toilet, or running water) was associated with a decreased likelihood of experiencing no damage (OR = 0.70, CI = 0.52–0.93). The predicted probability of no damage to the home for the top quartile of remittance receivers was 0.53 relative to 0.43 for those in the bottom quartile. If the main respondent had more than a high school education, the predicted the probability was 0.58 compared to 0.45 for those with less education. Those in the top income quartile showed a predicted probability of 0.53, relative to 0.48 for the third quartile and 0.43 for the bottom half. With regards to household amenities, those with one or no amenities showed a predicted probability of 0.43 relative to those with two or more with a 0.54 of not experiencing damage. Lastly, we summed the number of advantages (two or more amenities, top income quartile, higher than high school education and upper remittance quartile) and estimated the predicted probabilities based on a categorization of this sum. Those with three or more advantages showed a predicted probability of 0.60 compared to 0.49 for two, and 0.43 for one or less advantage.

### Predictors of Whether the Child Attended School

From Table 3, children who had been assaulted had lower odds of being enrolled in school, compared with children who had not been victimized (OR = 0.55, CI = .32–.95). The marginal, predicted probabilities of being in school were 0.23 for children who had been victimized, compared with 0.38 for those who had not been victimized prior to the earthquake. Children who had a chronic or acute illness were also much less likely to be attending school than other children, with predicted probabilities of attending school equal to 0.22 for those children who were ill, compared with 0.41 for children who were not ill. Girls were far less likely to attend schools than boys, (OR = .47, CI = .39–.56) as were children who had their own earned income (OR = .55, CI = .398–.760). No statistically significant association was observed for the relationship between age and the probability of attending school.

Increases in the log of household income (OR = 1.73, CI = 1.17–2.58) and the log of monetary gifts received by the household (OR = 1.55, CI = 1.44–1.67) were both associated with higher odds that a child in the household was attending school. The predicted marginal probability that a child in the household was attending school ranged from 0.17 for households in the lowest income quartile to 0.59 for households in the highest quartile. The odds that a child was attending school were also lower for children living in households with a higher percentage of household members who were chronically or acutely ill (OR = .25, CI = .11–.55). Odds of attending school were lower for children living in households in which the household respondent had less than a high school education, compared with more highly educated respondents and where the respondent reported that the level of insecurity and crime faced by the household was serious or very serious. The predicted probability that a child in a household with a serious to very serious level of reported insecurity and crime was 11 percentage points lower than for other children. Neither the number of children nor the number of adults in the household was associated with the probability that the child was attending school.

### Predictors of Going Hungry Modeled with and without Household Damage

Two separate specifications were assessed for the logistic regression analysis for the child going hungry after the quake (Table 4). In Column 1, the table shows the results of the regression without the inclusion of the household damage variable while Column 3 shows the results with that variable included. Using the Chow Test of Equality of Coefficients, the results show that the inclusion of the household damage variable did significantly alter the several of the coefficients in the model; there were no substantive changes in the odds ratios. With that, no household damage is significantly associated with a decreased likelihood of the child going hungry (OR = 0.54, CI = 0.35–0.84).

Reporting out with household damage in the model, children who were attending school prior to the earthquake had lower odds of experiencing food insufficiency following the earthquake, compared with children who had not been attending school (OR = 0.80, 95% CI = 0.48–0.85. Age, gender, and health prior to the quake of the child were not associated with going hungry after the quake.

As for household-level predictors, children who were living in households without any electricity, flush toilets or running water prior to the earthquake demonstrated a substantial increased likelihood of going hungry relative to having two or more amenities (OR = 8.19, CI = 3.56–18.82) as did those with only one amenity (OR = 2.75, CI = 1.28–5.91). The predicted probability of going hungry when the household had all three amenities was 0.04 and was 0.38 if the household did not have any of those amenities. The amount of remittances (ln) were significantly associated with a decreased likelihood of going hungry (OR = 0.80, CI = 0.66–0.95).

The health of family members prior to the quake also demonstrated an association with going hungry after the quake. The percentage of household members who were chronically/acutely ill prior to the quake showed a positive association with the likelihood of going hungry (OR = 4.97, CI = 1.76–14.05) as was the respondents' level of PTSD symptomology (OR = 1.51, CI = 1.09–2.09).

Personal security also demonstrated associations with reports of going hungry. If a member of the household was victimized prior to the quake and if the main respondent felt that the security situation was serious were both associated with an increased likelihood of hunger reporting (OR = 2.11, CI = 1.16–3.85 and OR = 2.20, CI = 1.12–4.34, respectively).

The number of householders was also associated with going hungry. The number of adults and the number of children were associated with a decrease likelihood of going hungry (OR = 0.85, CI = 0.77–0.93 and OR = 0.80, CI = 0.70–0.92, respectively).

### Predictors of Cutting the Size of Meals and Skipped Meals

We find that there are similar associations between household and child-specific variables and likelihoods of cutting meal sizes and skipping meals and children going hungry. On child-specific variables we find that children that went to school prior to the quake were significantly less likely to have had meal cut or skipped a meal (OR 0.75, CI = 0.57–0.98; OR 0.62 CI = 0.48–0.80, respectively). Examining Appendix V in File S1, the predicted probability of a child going hungry that was not in school was 0.20 (SE = 0.01), while it was 0.08 (SE<0.01) for those who had been in school. Age, gender, and acute/chronic illness were not significantly associated with the food security indices.

On household-level characteristics, having no or only one amenity (electricity, flush toilet, or running water) in the house prior to the earthquake was significantly associated with food insecurity on these two indices. Relative to having two or more of these amenities, those with no amenities were substantially more likely have cut or skipped a meal (OR 7.45, CI = 3.72–14.91; OR 10.25 CI = 4.90–21.41) as were those with only one amenity (OR 2.23 CI 1.18–4.22; OR 3.16 CI 1.63–6.11). The predicted probability of cutting or skipping meals was 0.48 (SE = 0.03) and 0.48 (SE = 0.02) respectively, while for those who had all three amenities the predicted probabilities were 0.18 (0.01) and 0.17 (SE 0.01), respectively. If the household did not have visible damage, children were significantly less likely to have skipped a meal (OR 0.58, CI = 0.39–0.85), but was not significant for cutting meals (p.<.10). The predicted probability (Appendix IV in File S1) of skipping a meal when the household was visibly damaged was 0.22 (SE = 0.01), while for no visible damage it was 0.17 (SE = 0.01).

While the log of household income was not significantly associated with cutting or skipping meals, the log of remittances were significantly associated with a decreased likelihood (Cutting Meals OR 0.71, CI = 0.58–0.87; Skipping Meals OR 0.81, CI = 0.69–0.95). The predicted probability (Appendix IV in File S1) of cutting meals when there was no remittances was 0.26 (SE = 0.01) and for skipping meals 0.25 (SE = 0.01). For those in the top quartile in the value of remittances, if any was received, the predicted probability of cutting a meal was 0.06 (SE = 0.01) and of skipping a meal 0.08 (SE = 0.01).

Perceptions of security were also significantly associated with the cutting and skipping meals. Those respondents who felt security was a serious issue pre-quake were more likely to have cut (OR 2.10, CI = 1.06–4.18) or skipped (OR 2.67, CI = 1.44–4.95). The predicted probability of cutting a meal when security was serious issue was 0.23 (SE = 0.01) and 0.07 (SE = 0.01) when it was not serious. With regards to skipping meals, the predicted probability (Appendix V in File S1) when security was considered serious was 0.23 (SE = 0.01) compared to 0.06 (SE<0.01) when it was not considered serious. Findings were similar if the main respondent had been assaulted prior to the quake for cutting meals (OR 2.23, CI = 1.32–3.72) but was not significant for skipping meals. The predicted probability of cutting meals if the respondent was assaulted was 0.44 (SE = 0.02) compared to 0.15 (SE = 0.01) if they were not.

The mental and physical health of the main respondent and/or the other householders prior to the quake was also shown to be associated with cutting and skipping meals. The percentage of householders who were chronically or acutely ill prior to the quake showed an odds ratio of 9.92 (CI = 3.80–25.86) for cutting meals and an odds ratio of 8.91 (CI = 3.16–25.11) for skipping meals. The predicted probability (Appendix V in File S1) of cutting a meal when 50% or more of the household was chronically/acutely ill was 0.60 (SE = 0.04) but when there was no one ill it was 0.10 (SE = 0.01). For skipping meals, the predicted probability was 0.58 (SE = 0.04) when over 50% of the householders were ill, compared to 0.11 (SE = 0.01) when no one was ill. The level of PTSD symptomology for the respondent was also associated with an increased likelihood of cutting or skipping meals. For cutting meals, the odds ratio for PTSD was 1.65 (CI = 1.24–2.20) and for skipping meals, 1.76 (CI = 1.31–2.35). The size of the household for both adults and children was significantly associated with cutting and skipping meals (OR 0.79, CI = 0.69–0.90; OR 0.87 CI 0.80–0.95, respectively).

In Appendix V in File S1 the analysis is stratified by whether the child was in school prior to the quake to access the extent to which being in school impacts the relative odds of child hunger for the other variables. This specification does demonstrate one statistically significant difference in coefficients between the two subgroups. Girls were also showed significantly decreased likelihood of going hungry when attending school relative to those who were not attending school. All other findings were determined to be equivalent.

## Discussion

This paper examined two specific types of vulnerabilities before and after the earthquake. We included analysis of school attendance prior to the quake as it is posited to be a significant potential protective factor on food insecurity after the quake and illuminates the vulnerability of children prior to the quake. Similar to past research on the differential impact of natural disasters by socio-economic status, our research findings also suggest the capabilities and functioning of the household prior to disaster were strongly related to negative outcomes regarding food insufficiency for children that were observed in Haiti following the earthquake. Overall the results indicated that many Haitian children experience multiple vulnerabilities that are likely to have major long term consequences for their physical, psychological and economic well-being. Furthermore children who were living in households experiencing the greatest economic and psychological vulnerabilities prior to the earthquake were also the children most likely to be experiencing food insufficiency following the earthquake.

### Schooling and Food Security

After the earthquake, those children who had not been in school prior to the earthquake were, in turn more, likely to experience different dimensions of food insecurity. When examining food security, two of the three food security measures were associated with school attendance. Beyond that condition, females, assault victims, living in a lower income household and working children are all associated with lower predicted probabilities of school attendance prior to the quake. Indeed, almost two-thirds of school-aged children were not enrolled in school prior to the quake.

Children in general were less likely to be food insecure after the quake if they were in school. The reasons for this finding may rest with the unmeasured characteristics of the adults in the household, pertaining to their underlying capabilities and functioning. The capabilities and set of functionings available to and achieved by adults (and parents) who encourage and seek educational opportunities for children may differ substantially from those available to and achieved by adults whose children do not attend school.

### The Experience of Violence

Our results strongly supported our hypothesis that violence and the direct experience of physical or sexual assault will diminish the capabilities and functionings of both children and adults in the household. Not only did we find that children who were not attending school were more likely themselves to have been victimized, but they also were more likely to have lived in households where the perceived levels of insecurity and crime were viewed as serious or very serious. Children who experienced food insufficiency were also more likely to be living in households that had already been victimized prior to the earthquake and in which adult members of the household had higher levels of post-traumatic stress, compared with children not experiencing food insufficiency.

### Income and Living Conditions

As noted above, income, particularly labor-market income, generally provides only an incomplete picture of the extent of poverty and economic well-being experienced by households. Our findings confirm the importance of making a distinction among different sources of income and observed living conditions. In our study, household living conditions were associated with food security, where having none or only one of the following amenities: electricity, flush toilets, or running water, was associated with significantly higher probabilities of being food insecure after the quake. Living conditions, even when controlling for all sources of income, seem to matter. This may be a result of the location of poorer housing. In particular, those households residing in the ‘popular zones,’ the densely packed, poorer areas of Port-au-Prince. in which one would expect to find fewer household amenities, were particularly underserved by humanitarian organizations in the immediate aftermath of the quake [21]. Those households were significantly more likely to have had their residence damaged or destroyed and were significantly more likely to be residing outside or in IDP arrangements [1]. These popular zones were also associated with poorer security conditions, [13] which may also explain why human rights victimization and perceptions of security prior to the quake was also associated with food insecurity in at least 2 out of the 3 categories.

With regards to housing damage as a result of the quake, we find that income, remittances, high school graduates were associated with an increased likelihood of having no visible damage as a result of the earthquake. Conversely, having only one of the aforementioned amenities was associated with an increased likelihood of having had damage.

Somewhat surprisingly, household income was only associated with cutting the size of the meals, while outside remittances were associated with reductions in all three measures of food security. This finding is consistent with Kolbe et al.'s [1] findings on remittances and food insecurity, although they did not control for household income in their analysis. Dollar-for-dollar, remittances were substantially more likely to reduce food insecurity than other sources of income. As noted above, it may be that remittances free up time to seek food basics, rather than having to find or earn income. Remittances also tend to provide recipients with greater autonomy in how the monies can be used, compared with resources or aid from governmental agencies or INGOs.

### Household Health

As hypothesized in our conceptual model, the percentage of the household that was chronically/acutely ill, whether the child suffered from a chronic/acute illness and the level of PTSD symptomology in the respondent were all associated with higher probabilities of food insecurity in at least 2 out of the 3 categories. The diminished capacity of adults and children who are ill to obtain food may explain these findings. The household itself might also face additional diminished capacity and functioning because healthy family members may be devoting additional time to caregiving that could otherwise be directed at obtaining resources to acquire food. Anecdotally, several respondents told survey investigators that the most able individuals were the ones that were most likely to receive aid as they could travel to aid locations and physically assert themselves at those locations.

### Limitations

Standard limitations for survey studies apply to this study. While the longitudinal nature of this dataset provides an opportunity to analyze post-earthquake outcomes as they relate to conditions prior to the earthquake, there are several caveats that should be considered. In the second wave of data collection in February 2010, the researchers were unable to locate or interview 3.8% of the original 1,800 households. It is possible that these households may have been disproportionately affected by the earthquake (e.g., the entire household had fled Port-au-Prince for food security reasons). In addition, those that refused to participate in 2009 may have been more or less susceptible to poor outcomes such as school dropout, human rights victimization, and overall poverty that could potentially alter our models. Given that we do not know any of the characteristics of non-responding households, it is not possible to correct statistically for this potential bias. However, the initial response of 90% in 2009 was high, so response bias is likely to be modest. Lastly, this study only represents the Port-au-Prince metropolitan area; we do not purport to report on experiences of those living outside of surveyed areas.

These findings depend on oral reports given by a household member about his/her own experiences, as well as the experiences of others in the household. It is possible that some events may not have been adequately reported. Some assault victims may have been reluctant to disclose their personal experiences with the interviewers, or may not have disclosed the event to the household member who was the primary respondent for the survey.

### Implications

Adult capabilities and functionings, particularly as they pertain to parenting practice and/or the wherewithal to secure food and other basic necessities after a natural disaster, may be associated with keeping their children in school. Identifying and providing the types of supports needed by parents so that they can ensure that children attend school, may have secondary positive effects on their capability to provide adequate food for their children. This recommendation is given with the caveat that substantially more research needs to be conducted to understand how parental capability and functioning can best be strengthened in the Haitian context.

It will be incumbent in future disaster relief efforts, in Haiti and in other insecure countries, that aid be targeted to areas that are often times avoided in the hopes of keeping aid workers safe from perceived threats. However, as the authors showed in previous work [1], although sexual assault and thefts were common, physical assaults were not, and victims were most likely those who were residing outside or in the IDP camps. Establishing security threats post-disaster and responding appropriately are of tantamount importance. Given that, there is often a difference between perceived and actual threats [22]. The popular zones suffered the greatest level of devastation in Port-au-Prince. It appears that the threat to workers after the quake was not as grim as had been assessed by the UN, and because of that misconception, aid was not provided where it was most needed. While we do not advocate for unduly putting aid workers in harm's way, we do suggest that an establishment of a solid understanding of the security situation in various sectors just after a disaster is a key piece of intelligence. Once established, aid delivery can proceed safely, based on real-time information.

Immigration policy in developed countries that specifically allows greater numbers of immigrants from very poor countries may create a mechanism for providing aid to families in the country of origin that may possibly circumvent some of the difficulties that have been attributed to formal foreign aid. In particular, it has been argued that formal consistent aid (foreign particularly) over the years and the massive influx of aid after the quake has potentially decreased the capacity of Haitians and the Haitian government to create the economic, social, and governmental systems necessary for sustainable development [23], [24]. Development and reconstruction experts must balance the need to help capacitate Haitians to develop sustainable systems, while, at the same time, respecting and responding to the need for immediate food assistance. By recognizing the role that immigration policy can play in the provision of aid through remittances, these experts can consider the possibility that a greater reliance on remittances may provide greater autonomy to Haitians in the development of these systems.

As noted earlier, there is a serious dearth of literature on the predictors of mortality and morbidity prior to a natural disaster. With that, it is often difficult to obtain randomized survey results after a natural disaster as many households will have relocated or the entire household was killed. Indeed, in Haiti, given the lack of information on the population, it was a challenge to conduct a rigorous representative survey prior to the quake. There is substantial literature on conducting surveys in difficult environments, such as refugee/IDP camps and other places were individuals have relocated. However, because of the nature of who occupies these camps, the samples from these camps are inherently biased and thus representativeness difficult to obtain. These findings suggest that carefully constructed surveys (i.e., a properly vetted survey instrument) with a randomized sample frame prior to the event can yield representative results that are extremely useful in a timely fashion. With longitudinal data of the population prior to these events, we can then begin to parse the antecedents to victimization to natural or human-made disasters. To accomplish this, we suggest that cohort studies, with an eye to vulnerabilities to victimization, be conducted in locations with high probabilities of disaster. This could lead to better targeted and more efficient delivery of essential services after these sorts of events.

## Supporting Information

File S1 Appendix I, Survey Instrument. Appendix II, Predicted Probabilities of Household Damage. Appendix III, Predicted Probabilities of Attending School. Appendix IV, Predicted Probabilities of Food Insecurity Variables by Characteristic. Appendix V, Testing Equality of Coefficients for Child Hungry, Skipped Meals, Cut Size of Meals, for Stratification by Child in School.
(DOCX)
