# Supplementary material for: Features of Child Food Insecurity after the 2010 Haiti Earthquake: Results from Longitudinal Random Survey of Households
Source: PLoS One. 2014 Sep 10;9(9):e104497. doi: 10.1371/journal.pone.0104497 (PMC4160193; doi:10.1371/journal.pone.0104497)
Supplement: File S1 — Appendix I, Survey Instrument. Appendix II, Predicted Probabilities of Household Damage. Appendix III, Predicted Probabilities of Attending School. Appendix IV, Predicted Probabilities of Food Insecurity Variables by Characteristic. Appendix V, Testing Equality of Coefficients for Child Hungry, Skipped Meals, Cut Size of Meals, for Stratification by Child in School. (DOCX) [file pone.0104497.s001.docx]

**Appendix I – Survey**

A1. Interviewer: _____ (LAST NAME – USE FALSE NAME)

A2. Today’s Date: _____

A3. Number of Adults in Household (Q1)

**We have some questions about the home where you lived on the day of the earthquake.**

B1. Where was the home located? Enter neighborhood code ______. (FIRST NAME))

B2. Is anyone currently living (sleeping, bathing, and cooking, etc) at the home? (Q2) [1]YES [2]NO

B3. Was your home (Q3)

(1) Totally destroyed ( eg, fell down totally, cannot be repaired even if you had the money to hire someone to fix it)

(2) No, it was not destroyed, but it was damaged (**if 2, go to B4**);

(3) No, it was not damaged that I could see.

**(If 1 or 3 skip to B5)**

B4. What exactly was damaged (specify part of the home): (1) cracks or bulges in walls; (2) cracks or bulges in foundation; (3) collapsed upper floor(s); (4) collapsed roof; (5) retaining walls collapsed or bulging; (Q4)

B4v2. (1) other – describe (2) Unable to determine

B5.Are there dead bodies still in your home? (Q5) (1)YES (2)NO

B6. If there are debris, have they been removed? (Q6)

(1) Totally cleared (2) Partially cleared (3) Not yet cleared

(4) Does not need to be cleared (5) there are no debris

B7. What type of home did you live in (choose the one that most fits the description)? (Q7)

(1) One story detached (2) Two+ story detached (3) One story with shared walls or roof

(4) two+ story with shared walls or roof (5) apartment building

B8. What material was your ceiling made of? **(Q8)**

(1) Concrete (2) Wood (3) Chaume/Grass/Palm tree leaves (4) Metal (3)

B9. What material were your walls made of? **(Q9)**

(1) Concrete (2) Brick (3) Wood (4) Metal

(5) Cardboard/Plastic (includes sheeting and tarps)

B10. What material was your floor made of? (Q10)

(1) Concrete (2) Stones/rocks (3) Brick (4) Dirt or compacted earth fill (5) Tile

B11. What kind of yard did(does) your home have? (Q11)

(1) Private yard (2) Shared yard (3) No yard.

B12. Right now, if you were to go back to your home, would it be accessible by road? (Q12)

(1) Yes, there is no debris blocking route (2) No, there are debris blocking route **OR**

(3) No, the home is located off an informal path or within the corridors (4) I don’t know

***We have some questions about food. Now I’m going to read you several statements that people have made about their food situation. For these statements, please tell me whether the statement was often true, sometimes true, or***

***never true for (you/your household)since the earthquake.***

**2. The first statement is “(I/We) worried whether (my/our) food would run out before (I/we) got**

**money to buy more.” Was that often true, sometimes true, or never true for (you/your**

**household) since the earthquake? (it will be two months since the earthquake, not one) (Q13)**

**[ 1] Often true [2 ] Sometimes true [3 ] Never true**

**3. “The food that (I/we) bought just didn’t last, and (I/we) didn’t have money to get more.” Was**

**that often, sometimes, or never true for (you/your household) since the earthquake? (Q14)**

**[ 1] Often true [2 ] Sometimes true [3 ] Never true**

**4. “(I/we) couldn’t afford to eat balanced meals.” That is, meals that have a variety of fruits, vegetables, grains and proteins like meat, milk or beans. Was that often, sometimes, or never true for**

**(you/your household) since the earthquake? (Q15)**

**[ 1] Often true [2 ] Sometimes true [3 ] Never true**

**5. “(I/we) relied on only a few kinds of low-cost food to feed (my/our) child/the children) because**

**(I was/we were) running out of money to buy food.” Was that often, sometimes, or never true**

**for (you/your household) since the earthquake? (Q16)**

**[ 1] Often true [2 ] Sometimes true [3 ] Never true [ 4] DK or Refused**

**[IF CHILDREN UNDER 18 IN HOUSEHOLD, ASK Q6; OTHERWISE SKIP TO Q8]**

**6. “(I/We) couldn’t feed (my/our) child/the children) a balanced meal, because (I/we) couldn’t**

**afford that.” Was that often, sometimes, or never true for (you/your household) in the last**

**month? (A balanced meal is one that has a variety of fruits or vegetables, grains such as rice or wheat and proteins like meat, milk or beans.) (Q17)**

**[ 1] Often true [2 ] Sometimes true [3 ] Never true**

**[IF CHILDREN UNDER 18 IN HOUSEHOLD, ASK Q7; OTHERWISE SKIP TO Q8]**

**7. "(My/Our child was/The children were) not eating enough because (I/we) just couldn't afford**

**enough food." Was that often, sometimes, or never true for (you/your household) since the earthquake. (Q19)**

**[ 1] Often true [2 ] Sometimes true [3 ] Never true**

**8.Since the earthquake, did (you/you or other adults in your**

**household) ever reduce the size of your meals or skip meals because there wasn't enough money for**

**food? (Q21)**

**[ 1] Yes [2 ] No (SKIP 8a)**

**8a. [IF YES ABOVE, ASK] How often did this happen---almost every day, some days but**

**not every day, or in only 1 or 2 ? (Q22)**

**[ 1] Almost every day [ 2] Some days but not every day [ 3] Only 1 or 2 days**

**9. Since the earthquake,, did you ever eat less than you felt you should because there wasn't**

**enough money to buy food? (Q23)**

**[ 1] Yes [ 2] No**

**10. Since the earthquake, were you every hungry but didn't eat because you couldn't afford enough**

**food? (Q24)**

**[ 1] Yes [ 2] No**

**11. Since the earthquake, did you lose weight because you didn't have enough money for food? (Q25)**

**[ 1] Yes [ 2] No**

**12. Since the earthquake, did (you/you or other adults in your household) ever not eat for a whole**

**day because there wasn't enough money for food? (Q26)**

**[ 1] Yes [ 2] No (SKIP 12a)**

**12a. [IF YES ABOVE, ASK] How often did this happen---almost every day, some days but**

**not every day, or in only 1 or 2 days? (Q27)**

**[ 1] Almost every day [ 2] Some days but not every day [ 3] Only 1 or 2 days**

**[IF CHILDREN UNDER 18 IN HOUSEHOLD, ASK 13-16; OTHERWISE SKIP TO END.]**

**13. The next questions are about children living in the household who are under 18 years old. In the**

**lSince the earthquake, did you ever cut the size of (your child's/any of the children's) meals because there wasn't enough money for food? (Q28)**

**[1 ] Yes [2 ] No**

**14. Since the earthquake, did any of the children in your household ever skip meals because there wasn't enough money for food? (Q29)**

**[ 1] Yes [ 2] No (SKIP 14a)**

**14a. [IF YES ABOVE ASK] How often did this happen---almost every day, some days but**

**not every day, or in only 1 or 2 days? (Q30)**

**[ 1] Almost every day [ 2] Some days but not every day [ 3] Only 1 or 2 days**

**15. Since the earthquake, (was your child/ were the children) ever hungry but you just couldn't afford more food? (Q31)**

**[ 1] Yes [ 2] No**

**16. Since the earthquake, did (your child/any of the children) ever not eat for a whole day because**

**there wasn't enough money for food? (Q32)**

**[1 ] Yes [ 2] No**

AD4. What has been your main source of food for the past week? (Q33)

(1) Food we have stored (2) We go looking for food in damaged buildings or other places

(3) Relief agencies/charity gives us food (4) We buy food (5) We ask friends or neighbors for food.

W1. What is your water source? (Q34)

(1) Formal cistern (2) Water kiosk (3) Truck water delivered to a temporary cistern

(4) Buy bottled (5) From relief agency

W2. Do you have a place to store water? (Q35)

(1) Yes, one bucket or smaller (2) Yes, more than one bucket (3) Yes, drum(s)

(4) Yes, tank or cistern (5) No

W3. Is water that you use for drinking and cooking is clean/treated? (specify) (Q36)

(1) Yes, we treat it (2) Yes, others treat it (3) No, not treated.

W4. Do you have access to a bathtub, shower or other place to bathe? (Q37)

(1)YES **(If ‘yes’ go to W5)** (2)NO **(If ‘no’ skip to S1)**

W5. If yes, is it private? (Q38) (1)YES (2)NO

**Shelter Facilities**

S1. What toilet facilities are you using right now? **(If filled then skip to S7) (Q39)**

(1) pit toilet shared with others (2) pit toilet for our household only (3) flush toilet that we share with others; (4) flush toilet that is for our household;

(5)we don’t have toilet facilities and just go wherever we find a place to go.

S7. Right now, when you have garbage, where do you get rid of it? **(If filled then skip to S8) (Q40)**

(1) Open container (2) Closed container (3) Dump in ravine

(4) Trash pile on the street (5) By burning

S8. Where did you sleep last night? **(If filled then skip to S9) (Q41)**

(1) In a tent (2) In a building (3in a car

(4) Under a tarp (5) In the outdoors/under the sky

S9. Where is your sleeping location? **(If filled then skip to S10) (Q42)**

(1) on our land (2) family home/land (3) a friend’s home/land

(4) a community place (church, school yard) (5) open land

S9v2. (Q43) (1) organized camp (2) informal camp (3) a public place (plaza)

(4) other, describe

**Policing & Community Services**

C1. In the area where you are staying right now, when was the last time you saw police (HNP) either on patrol or otherwise working in your area? (Q44)

(1)Today (2)Yesterday (3)The past week (4) 14-28 days ago

(5) I have not seen them since the earthquake

C2. In the area where you are staying right now, when was the last time you saw foreign troops on patrol or otherwise working in your area? (Q45)

(1)Today (2) Yesterday (3)The past week (4) 14-28 days ago

(5) I have not seen them since the earthquake

**We are interested in knowing how you would respond to some problems that people have. If you were confronted with any of these problems right now, who would you go to for help?**

P1) Right now, if you had a land or property dispute, who would you turn to? **(Q46)**

(1)Local government (2)National government or ministry (3) Police (4)Lawyer/Courts/Judge

(5)Ask a community leader for help

P2) Right now, if you needed to obtain a government document, who would you turn to?  (Q47)

(1) Government ministry (2) Mayor’s office (3) Go to a community leader or NGO for help

(4) Other (5) I don’t know

P3) (If person says “government ministry” for question above, ask) If you had no success obtaining the document when you went to the government ministry to get it, who would you turn to for help or what would you do next? (Q48)

1. Turn to relative/friend/neighbor for help
2. Go to police.
3. Go to an NGO for help.
4. Go to the foreign military or UN.
5. Turn to community elders.

P4) If there was a personal dispute in your family involving money, who would you turn to for help?  (Q49)

1. Turn to relative/friend/neighbor for help
2. Go to police.
3. Go to the foreign military.
4. Go to private security company or similar.
5. Turn to community elders.

P5) If there was a dispute in your family and someone in your family hurt another family member, who would you turn to for help? (Q50)

1. Turn to relative/friend/neighbor for help
2. Go to police.
3. Go to the foreign military.
4. Go to private security company or similar.
5. Turn to community elders.

P6) If there was a great deal of discord and arguing within your family, who would you turn to for help? (Q51)

1. Friend
2. Neighbor
3. Family member
4. Community or Church Leader
5. Police

P7) If you or a family member had a minor legal problem, who would you turn to for help? (Q52) (Shorten?)

1. Turn to relative/friend/neighbor for help
2. Go to police.
3. Go to an NGO for help.
4. Go to the foreign military or UN.
5. Lawyer/Courts/Judge

P8) If you have asked for help with a minor legal problem in the past, who did you turn to? (Q53) (Shorten?)

1. Turn to relative/friend/neighbor for help
2. Go to police.
3. Go to an NGO for help.
4. Go to the foreign military or UN.
5. Lawyer/Courts/Judge

P9) What is your opinion of the assistance that you received for this minor legal problem? (Q54)

1. the assistance I received was the very best assistance possible

2. the assistance I received was good, it was more than adequate but still could have been better

3. the assistance I received was adequate, but nothing outstanding

4. the assistance I received was not very good at all

5. the assistance I received was very bad

P10) Did you pay for the assistance that you received in dealing with this minor legal problem? (Q55)

(1)Yes (2) No

P11) Are there clear procedures for passing down land/property from father to son in your family? (Q56)

(1)Yes (2) No

P12) Are there are clear procedures for passing down land/property from father to daughter in your family? (Q57)

(1)Yes (2) No

P13) Did you on your spouse/partner receive any land/property from your father/mother in your lifetime? (Q58)

(1)Yes (2) No

__ Local council officials

__ Community leaders

__ Church

__ The child should become a restavek and be the responsibility of the family they live with

__ Foreign NGOs

__ Haitian NGOs

**Q) I am going to list several problems that people sometimes have. Please indicate whether you think they are a very serious (1), serious (2), moderate (3) or minor (4), very minor (5) problem? Please compare your views now with before the earthquake.**

| Problem | Before earthquake | Now |
| --- | --- | --- |
| 01. Unemployment | (Q59) | (Q60) |
| 02. Insecurity or crime | (Q61) | (Q62) |
| 03. Capacity of government | (Q63) | (Q64) |
| 04. Access to/quality of medical services | (Q65) | (Q66) |
| 05. Access to/quality of educational services | (Q67) | (Q68) |
| 06. Corruption in the public sector | (Q69) | (Q70) |
| 07. Corruption in the private sector | (Q71) | (Q72) |
| 08. Difficulties finding housing or shelter | (Q73) | (Q74) |
| 09. Availability of drinking water | (Q75) | (Q76) |
| 10. Availability of electricity | (Q77) | (Q78) |
| 11. Availability of food | (Q79) | (Q80) |
| 12. Availability of compensation for property losses due to emergencies such as floods, earthquake or violence | (Q81) | (Q82) |
| 13. Access to information about government services | (Q83) | (Q84) |

R1) Have you or your household received any services since the earthquake from the following:

R2) And if yes, what was the quality of services you received?)

a - the assistance I received was the very best assistance possible

b - the assistance I received was good, it was more than adequate but still could have been better

c - the assistance I received was adequate, but nothing outstanding

d - the assistance I received was not very good at all

e - the assistance I received was very bad

|  | Services | Quality |
| --- | --- | --- |
| 01. Local government (e.g. mayor’s office) | (Q85) | (Q86) |
| 02. Ministry of the national government | (Q87) | (Q88) |
| 03. Publically run community service (hospital, school, Teleco, CAMEP, etc) | (Q89) | (Q90) |
| 04. International NGO | (Q91) | (Q92) |
| 05. Haitian NGO | (Q93) | (Q94) |
| 06. Local community group that is not an NGO (e.g., trade union, women’s group, political group, etc) | (Q95) | (Q96) |

**R3) I am going to cite several priorities for you and your household over the next 3 months. Please rank them in order of priority with 1=top priority.**

| Priority | Rank |
| --- | --- |
| 01. finding a job | (Q97) |
| 02. having a place to live | (Q98) |
| 03. repairing my property | (Q99) |
| 04. Holding the funeral my family member(s) | (Q100) |
| 05. Being able to deal with the hurricane season | (Q101) |

**R4) I am going to cite several more priorities for you and your household over the next 3 months. Please rank them in order of priority with 1=top priority.**

| Priority | Rank |
| --- | --- |
| 01. Having access to water | (Q102) |
| 02. Managing crime/insecurity | (Q103) |
| 03. Access to medical care | (Q104) |
| 04. Access to education | (Q105) |
| 05. Ensure relief funding is well spent | (Q106) |
| 06. Having access to electricity | (Q107) |
| 07. Having access to food | (Q108) |
| 08. Having access to the food I like to eat | (Q109) |
| 09. Availability of compensation for losses | (Q110) |
| 10. Access to information from the government and community leaders | (Q111) |

**Please tell me if the following statements are true or not true.**

T1. I can hold the government responsible for doing the work that the government is supposed to do.

T2. The government cares about my opinions.

T3. Civil servants take into consideration the opinion of people like me when making decisions

T4. If I paid an informal fee I would receive better service from the government.

T5. Right now, if you wanted to communicate with the local government regarding about an important need in your community, how would you do so? (Q112)

1. I would go to the office and request a meeting in person.
2. I would locate someone who works with the local government and go to them, in hopes that they would be able to do something.
3. I would call the office.
4. I would contact someone I know who is influential or connected and ask them to convey my message to those in power.
5. I would call a radio station and tell them what I wanted to say. (IF T5 FILLED SKIP TO T6)

T5v2. (SKIP IF T5 FILLED) (Q113)

1. I would organize or go to a demonstration (protest).
2. I would send a text message or use twitter.
3. Other (specify)
4. There is no way for me to communicate with the local government.
5. It doesn’t matter, the local government doesn’t respond anyway.

T6. Right now, if you wanted to communicate with the national government regarding an important need in your community, how would you do so? (IF T6 FILLED SKIP TO T7) (Q114)

1. I would go to the office and request a meeting in person.
2. I would locate someone who works with the national government and go to them, in hopes that they would be able to do something.
3. I would call the office.
4. I would contact someone I know who is influential or connected and ask them to convey my message to those in power.
5. I would call a radio station and tell them what I wanted to say.

T6V2. (SKIP IF T6 IS FILLED) (Q115)

1. I would organize or go to a demonstration (protest).
2. I would send a text message or use twitter.
3. Other (specify)
4. There is no way for me to communicate with the national government.
5. It doesn’t matter, the national government doesn’t respond anyway.

T7. Right now, if you wanted to communicate with the international organizations working in Haiti about an important need in your community, how would you do so? (IF T7 FILLED SKIP TO T8) (Q116)

1. I would go to the office and request a meeting in person.
2. I would locate someone who works with an international organization and go to them, in hopes that they would be able to do something.
3. I would call the office.
4. I would contact someone I know who is influential or connected and ask them to convey my message to those in power.
5. I would call a radio station and tell them what I wanted to say.

T7v2. (SKIP IF T7 IS FILLED) (Q117)

1. I would organize or go to a demonstration (protest).
2. I would send a text message or use twitter.
3. Other (specify)
4. There is no way for me to communicate with international organizations.
5. It doesn’t matter, the international organizations don’t respond anyway.

T8. How do you receive information on national news? (Q118)

1. Friends/Family
2. Talking with people I don’t know (eg, while on the street or on a taptap)
3. TV
4. Radio
5. Text message/twitter

T9. How do you receive information on relief delivery (shelter, food, water, medical services)? (Q119)

1. Friends/Family
2. Talking with people I don’t know (eg, while on the street or on a taptap)
3. TV
4. Radio
5. Text message/twitter

T10. How do you receive information on reconstruction projects in your area? (Q120)

1. Friends/Family
2. Talking with people I don’t know (eg, while on the street or on a taptap)
3. TV
4. Radio
5. Text message/twitter

T11. Right now, how does government communicate with you? (Q121)

1. Radio
2. TV
3. billboard/posters/signs
4. other (specify)
5. Government doesn’t communicate with me

H1. How many people lived in your household on the day of the earthquake? (**DAY**)

H2. How old are you? (**YEAR** for respondent)

H3v2. How many people were killed in this household as a result of the earthquake?

**(IDENTIFICATION NUMBER ROW A)**

H4v2. How many people were seriously injured in this household?

**(IDENTIFICATION NUMBER ROW B)**

H4v3. How many people were sustained minor injuries in this household?

**(IDENTIFICATION NUMBER ROW C)**

H4v4. How many children were seriously injured in this household?

(IDENTIFICATION NUMBER ROW D)

H5v2. Of the seriously injured, how many received medical care from a professional caregiver?

H5v3. Of the seriously injured children, how many received medical care from a professional caregiver?

(IDENTIFICATION NUMBER ROW E)

H6. Was the care that your household received adequate? (Q122) (1)YES (2)NO

H8. How many people in the household have been seriously ill since the earthquake?

(INDENTIFICATION NUMBER ROW F)

H9. How many of the seriously ill received adequate treatment for their illness? (Q123)

H10v2. What is the respondent’s gender? (GENDER) (1) Male (2) Female

H12v2. How many years of full-time education has the respondent completed? (Q124)

NOTE: 13 years = High School Graduate

H14v2. What is the employment status of the respondent? (Q125)

NOTE: If unemployed and not looking for a job leave blank.

H15v2. How much did the household receive in the past month in cash for working? (Q126)

(1) 1-50 dollars (2) 51-100 dollars (3) 101-200 dollars (4) 201 -500 dollars

(5) 501 or more dollars.

H16v2. How much did the household receive in the past month in cash from gifts? (Q127)

(1) 1-50 dollars (2) 51-100 dollars (3) 101-200 dollars (4) 201 -500 dollars

(5) 501 or more dollars.

H17. Where did this money come from? (Q128)

(IF “OTHER” LEAVE BLANK ON SCANTRON)

1. A family member in Haiti
2. A friend in Haiti
3. A family member abroad
4. A friend abroad
5. Church/Charity

H18v2. What is the religion of the respondent? (Q129)

(1) Catholic (2) Catholic and Voodoo (3) Only Voodoo (4) Christian (other)

(5) Other, not Christian or Voodoo

H21) Since the earthquake, have you become: (Q130)

(1) more religious (2) about the same (3) less religious than before?

H25) Since the earthquake, how many times has your property or property belonging to someone in your household been stolen? (Q131)

(1) One (2) Two (3) Three (4) Four or more (5) None

H27) Since the earthquake have you or any household member been held against your will, that is, arrested, detained or kidnapped, by the police, an armed group, the military, criminals or anyone else? (Q132) (1)YES (2) NO

**IF ‘NO’ SKIP TO H28**

H27v2. How many total times has this happened to your family since the earthquake? (Q133)

(1) One (2) Two (3) Three (4) Four (5) Five or more times

H28) How many times in the past year has someone threatened to kill you or hurt you physically? (Q133)

(1) One (2) Two (3) Three (4) Four or more (5) None

H29) How many times since the earthquake has someone threatened to kill you or hurt you physically? (Q134)

(1) One (2) Two (3) Three (4) Four or more (5) None

H31) Since the earthquake, has anyone attacked you or a member of your household physically? *Physical attacks are incidents where someone mistreats another person causing serious injury to their body.* (Q135) (1) YES (2)NO

*Now, changing subjects for a moment, I’d like to ask about your emotions during the past week. The following are problems that people sometimes have after experiencing hurtful or terrifying events. I’m going to read each one to you and I’d like you to decide how much the problems bothered you in the past week.*

**1 – not at all 2 – a little 3 – quite a bit 4 – extremely**

x59. Recurrent thoughts or memories of hurtful or terrifying events. (Q136)

x60. Feeling as though the hurtful or terrifying event is happening again(Q137)

x61. Recurrent nightmares(Q138)

x62. Feeling detached or withdrawn from people(Q139)

x63. Unable to feel emotions (Q140)

x64. Feeling jumpy, easily startled (Q141)

x65. Difficulty concentrating(Q142)

x66. Trouble sleeping(Q143)

x67. Feeling on guard(Q144)

x68. Feeling irritable or having angry outbursts(Q145)

x69. Avoiding activities that remind you of the traumatic or hurtful event. (Q146)

x70. Inability to remember parts of the most traumatic or hurtful event. (Q147)

x71. Less interest in daily activities(Q148)

x72. Feeling as if you don’t have a future (Q149)

x73. Avoiding thoughts or feelings associated with the experience(Q150)

x74. Sudden emotional or physical reaction when reminder of the event(Q151)

People have different reactions to difficult events in their lives. Comparing now to the time before January 12^th^, please tell me how true these statements are for you:

**1 – not at all 2 – a little 3 – quite a bit 4 – extremely**

z1. I have new opportunities which would not have been available otherwise. (Q152)

z2. I accept better the way things turn out. (Q153)

z3. I can do more good with my life. (Q154)

z4. I know that I can deal with problems better. (Q155)

z5. I discovered that I am stronger than I thought I was. (Q156)

z6. I established a new path for my life. (Q157)

z7. I have more self-confidence. (Q158)

z8. I understand spiritual matters better. (Q159)

z9. I am more willing to express my feelings. (Q160)

Z10. I appreciate the value of my life more. (Q161)

Z11. I appreciate each new day more. (Q162)

Z12. I try to have the best relationships to others. (Q163)

Z13. I developed new interests. (Q164)

Z14. I know that I can count more on people when I am in trouble. (Q165)

Z15. I have more compassion for others. (Q166)

Z16. I try more to change things which need changing. (Q167)

Z17. I accept more that I need other people. (Q168)

Z18. I learned a lot about how wonderful people are. (Q169)

Z19. I believe more strongly in God. (Q170)

Z20. I feel closer to others. (Q171)

Z21. My aims in life changed. (Q172)

**160A)** Who is the first person that you would address/call, if an important asset belonging to you were robbed? (Q173)

1. Turn to relative/friend/neighbor for help
2. Go to police.
3. Go to former members of Haitian Army.
4. Go to the foreign military.
5. Go to private security company or similar. (IF FILLED OUT SKIP TO 161A)

**161B (DO NOT FILL IF 160A IS FILLED OUT) (Q174)**

1. Turn to community elders.

2. Turn to the head of the family.

3. Other: (specify) ____________

4. Nothing/no point in doing anything.

5. Don’t know.

**161A)** Who is the first person you would address/call, if someone threatened to hurt or kill you? (Q175)

1. Turn to relative/friend/neighbor for help

2. Go to police.

3. Go to former members of Haitian Army.

4. Go to the foreign military.

5. Go to private security company or similar. (IF FILLED OUT SKIP TO 161A)

**161B) (DO NOT FILL IF 161A IS FILLED OUT) (Q176)**

1. Turn to community elders.

2. Turn to the head of the family.

3. Other: (specify) ____________

4. Nothing/no point in doing anything.

5. Don’t know

**162A)**  Ideally, who do you think should be responsible for security? (Q177)

1. Local government
2. Ministry of Interior
3. Army
4. Former members of armed forces
5. Private security firms

**162B) (DO NOT FILL IF 162A IS FILLED) (Q178)**

1. The community

2. Family

3. The UN/foreign military

4. Other: (specify) __________

5. Don’t know.

*We’re almost done. Now I’m going to ask you more about your opinions regarding security. Please tell me whether you think that strongly agree, agree, disagree or strongly disagree with the following:*

Strongly Agree (1) Agree(2) Disagree(3) Strongly Disagree(4) DK(5)

**172)** Improve the capacity of police/security services would make my community safer. (Q179)

**173)** Greater control of legal licenses for firearms would make my community safer. (Q180)

**174)** Harsher punishment for illegal weapons possession would make my community safer. (Q181)

**175)** Collecting illegal guns from their owners would make my community safer. (Q182)

**176)** Outlawing armed groups would make my community safer. (Q183)

**177)** Increased United Nations presence in Haiti would make my community safer. (Q184)

**179)** Peace Accords between armed gangs will make my community safer. (Q185)

**180)** The MINUSTAH troops should leave Haiti right away. (Q186)

**181)** The MINUSTAH troops commit violent acts against unarmed civilians. (Q187)

**182)** MINUSTAH soldiers are making my community safer. (Q188)

**183)** Jean Bertrand Aristide should be allowed to return to Haiti**. (**Q189**)**

**184)** In the past, supporters of Jean Bertrand Aristide committed a lot of violence against civilians. (Q190)

**185)** After the departure of Aristide, his supporters were targeted for violence by the interim Haitian government of Boniface Alexandre. (Q191)

**186)** Do anyone in your household own a gun? (Q192) (1) Yes (2) No

*Thank you for taking so much time to talk to me. I just have a few more questions. We’ve spoke about a lot of things today: the people you live with, the work that you do, your education, and a lot about things that you have seen, experienced and felt in the past year. The information you are giving us today will help people understand what your community has experienced since 2004. While this information is very important, please remember that you don’t have to answer any questions that you don’t want to and you can stop this interview at any time. Okay?*

196A) Earlier I asked you about threats of physical violence and death threats. Now I’m wondering if, since 1 January 2004, anyone has threatened you sexually. That is, threatened with sexual contact that you did not want to have. How many times has this happened to you since 1 January 2004?

196B) Since the earthquake, has anyone threatened you sexually? (Q193) (1)YES (2) NO

107B) Since the earthquake, did anyone force you or another person in your household to have sexual contact that wasn’t wanted? (Q193) (1) YES (2) NO

107B – v2 Since the earthquake, how many times in total have household members been forced to have sexual contact they did not want to? (Q194)

(1) One (2) Two (3) Three (4) Four (5) Five or more

198) During your childhood, were you ever touched sexually by an adult or an older teen? (Q195)

(1) YES (2) NO

**Appendix II – Predicted Probabilities of Household Damage**

| Predicted Probabilities that the Child’s House Had No Visible Damage | | | |
| --- | --- | --- | --- |
| Number of Amenities: Electricity, Flush Toilet, Running Water | | Predicted Probability | Standard Error |
| None or One | 0.54 | | 0.004 |
| Two to Three | 0.43 | | 0.002 |
| Top Income Quartile |  | |  |
| No | 0.44 | | 0.002 |
| Yes | 0.52 | | 0.003 |
| Respondent Had More than a High School Education |  | |  |
| No | 0.45 | | 0.002 |
| Yes | 0.58 | | 0.009 |
| Quartile for Ln gifts |  | |  |
| 1^st^ or 2^nd^ Quartile | 0.43 | | 0.002 |
| 3^rd^ Quartile | 0.48 | | 0.012 |
| 4^th^ Quartile | 0.53 | | 0.003 |
| Number of Above Advantages |  | |  |
|  |  |  |  |
| None or one | 0.43 | | 0.003 |
| Two | 0.49 | | 0.002 |
| Three or Four | 0.60 | | 0.004 |

**Appendix III – Predicted Probabilities of Attending School**

| Predicted Probabilities that a Child or Adolescent was Attending School | | |
| --- | --- | --- |
|  | No | Yes |
| Child was victimized | 0.38 | 0.23 |
| Child had chronic or acute illness | 0.41 | 0.22 |
| Child was female | 0.45 | 0.30 |
| Child had earned income | 0.39 | 0.19 |
| Household respondent had less than a high school education | 0.41 | 0.35 |
| Household respondent rated level of insecurity as serious | 0.46 | 0.35 |
| Household respondent suffered from post-traumatic stress | 0.38 | 0.32 |
| Income Quartiles |  | |
| Lowest quartile | 0.17 | |
| 2^nd^ quartile | 0.27 | |
| 3^rd^ quartile | 0.41 | |
| Highest quartile | 0.59 | |

**Appendix IV - Predicted Probabilities of Food Insecurity Variables by Characteristic**

|  | Predicted Probability | Standard Error | Predicted Probability | Standard Error | Difference in Predicted Probability |
| --- | --- | --- | --- | --- | --- |
|  | Not in School | | In School | |  |
| Child Was Hungry | 0.20 | 0.01 | 0.08 | 0.00 | 0.12 |
| Child Skipped Meals | 0.26 | 0.01 | 0.11 | 0.01 | 0.15 |
| Cut Size of Children’s Meals | 0.26 | 0.01 | 0.11 | 0.01 | 0.15 |
|  | House Had Visible Damage | | House Did Not Have Visible Damage | |  |
| Child Was Hungry | 0.17 | 0.01 | 0.12 | 0.01 | 0.05 |
| Child Skipped Meals | 0.22 | 0.01 | 0.17 | 0.01 | 0.05 |
| Cut Size of Children’s Meals | 0.22 | 0.01 | 0.18 | 0.01 | 0.03 |
|  | No Electricity, Flush Toilet, or Running Water | | House had Electricity, Flush Toilet, and Running Water | |  |
| Child Was Hungry | 0.38 | 0.02 | 0.04 | 0.00 | 0.34 |
| Child Skipped Meals | 0.48 | 0.02 | 0.05 | 0.00 | 0.44 |
| Cut Size of Children’s Meals | 0.48 | 0.03 | 0.06 | 0.01 | 0.42 |
|  | Lowest Income Quartile | | Top Income Quartile | |  |
| Child Was Hungry | 0.26 | 0.02 | 0.05 | 0.00 | 0.21 |
| Child Skipped Meals | 0.34 | 0.02 | 0.06 | 0.01 | 0.28 |
| Cut Size of Children’s Meals | 0.36 | 0.02 | 0.06 | 0.00 | 0.30 |
|  | No Remittances | | Top Quartile: Value of Remittances | |  |
| Child Was Hungry | 0.19 | 0.01 | 0.05 | 0.01 | 0.14 |
| Child Skipped Meals | 0.25 | 0.01 | 0.08 | 0.01 | 0.17 |
| Cut Size of Children’s Meals | 0.26 | 0.01 | 0.06 | 0.01 | 0.20 |
|  | Household was victimized | | Household was not victimized | |  |
| Child Was Hungry | 0.34 | 0.02 | 0.11 | 0.00 | 0.23 |
| Child Skipped Meals | 0.41 | 0.02 | 0.16 | 0.01 | 0.25 |
| Cut Size of Children’s Meals | 0.44 | 0.02 | 0.15 | 0.01 | 0.29 |
|  | Insecurity Serious | | Insecurity Not Serious | |  |
| Child Was Hungry | 0.18 | 0.01 | 0.05 | 0.00 | 0.13 |
| Child Skipped Meals | 0.23 | 0.01 | 0.06 | 0.00 | 0.18 |
| Cut Size of Children’s Meals | 0.23 | 0.01 | 0.07 | 0.01 | 0.17 |
|  | No household members were acutely ill | | 50% or more of household members were acutely ill | |  |
| Child Was Hungry | 0.09 | 0.01 | 0.44 | 0.04 | -0.35 |
| Child Skipped Meals | 0.11 | 0.01 | 0.58 | 0.04 | -0.47 |
| Cut Size of Children’s Meals | 0.10 | 0.01 | 0.60 | 0.04 | -0.50 |

**Appendix V –**

**Testing Equality of Coefficients for Child Hungry, Skipped Meals, Cut Size of Meals, for Stratification by Child in School**

|  | (1)  Child Hungry  Not in School  Odd Ratio | (2)  Child Hungry  In School  Odds Ratio | Chow Test of Equality of Coefficients  Column 1 – Column 2 |
| --- | --- | --- | --- |
|  |  |  |  |
| VARIABLES |  |  |  |
|  |  |  |  |
| Age of Child | 0.99 | 0.92* | chi2(1)=2.79 |
|  | (0.02) | (0.03) | Prob>chi2=0.0947 |
| Female | 1.07 | 0.59+ | chi2(1)=4.01 |
|  | (0.15) | (0.16) | Prob>chi2=0.0453 |
| Child suffered from chronic/acute illness pre earthquake | 0.90 | 0.60 | chi2(1)=0.91 |
|  | (0.12) | (0.23) | Prob>chi2=0.3407 |
| Log of Household Income | 1.02 | 0.79+ | chi2(1)=2.47 |
|  | (0.13) | (0.10) | Prob>chi2=0.1159 |
| No_electricity_toilet_pipes | 7.59*** | 9.00*** | chi2(1)=0.09 |
|  | (3.40) | (5.45) | Prob>chi2=0.7601 |
| One_electricity_toilet_pipes | 2.75* | 2.72+ | chi2(1)=0.00 |
|  | (1.11) | (1.58) | Prob>chi2=0.9815 |
| Log of Monetary Remittances Received by Household | 0.76* | 0.85 | chi2(1)=0.69 |
|  | (0.09) | (0.09) | Prob>chi2=0.4045 |
| Percentage of household members who were chronically/acutely ill | 3.94* | 10.42** | chi2(1)=1.19 |
|  | (2.18) | (8.98) | Prob>chi2=0.2747 |
| Number of children in household | 0.87** | 0.77*** | chi2(1)=3.02 |
|  | (0.05) | (0.05) | Prob>chi2=0.0823 |
| Number of adults in household | 0.75*** | 0.95 | chi2(1)=5.13 |
|  | (0.06) | (0.09) | Prob>chi2=0.0235 |
| Household respondent level of PTSD symptoms | 1.38+ | 2.11** | chi2(1)=3.04 |
|  | (0.24) | (0.49) | Prob>chi2=0.0814 |
| Household member was victimized pre earthquake | 2.21* | 1.91 | chi2(1)=0.12 |
|  | (0.69) | (0.83) | Prob>chi2=0.7287 |
| Household respondent viewed level of insecurity as serious pre earthquake | 2.31* | 2.16 | chi2(1)=0.02 |
|  | (0.87) | (1.07) | Prob>chi2=0.8890 |
| House had no visible damage following earthquake | 0.64+ | 0.29*** | chi2(1)=4.98 |
|  | (0.15) | (0.10) | Prob>chi2=0.0257 |
| Constant | 0.06** | 0.09* |  |
|  | (0.05) | (0.11) |  |
|  |  |  |  |
| Observations | 2,184 | 1,412 |  |
| r2_p | 0.206 | 0.301 |  |
| p | 0 | 0 |  |
| chi2 | 100.2 | 103.8 |  |
| df_ | 14 | 14 |  |
| N_clust | 1018 | 758 |  |
